# Epidemiology and clinical characteristics of the Measles outbreak in the Nylon Health District, Douala Cameroon: a retrospective descriptive cross sectional study

**Published:** 2012-11-26

**Authors:** Gerald Etapelong Sume, André Arsène Bita Fouda, Marie Kobela, Salomé Nguelé, Irène Emah, Peter Atem, Daddy Mbida, Kondé Njock

**Affiliations:** 1 Regional Delegation of Public Health for the Littoral Region, Cameroon; 2 The Central Technical Group for the Expanded Programme on Immunization, Cameroon; 3 Nylon Health District Service, Cameroon

**Keywords:** Measles, line listing, outbreak, Cameroon, Nylon Health District

## Abstract

**Introduction:**

Measles is a public health problem especially in South Asia and Africa. Nylon Health District has experienced two measles outbreaks over a period of three years. We hereby describe the epidemiology and clinical characteristics of the outbreak of February 2011.

**Methods:**

A retrospective descriptive cross sectional study was conducted in November 2011. All suspected measles cases according to the World Health Organization case definition line listed in the district service were included. Data was analyzed using Epi Info version 3.5.3 for Windows and Microsoft Office Excel 2010. An epidemic curve was drawn and proportions per variable category were estimated and presented in frequency tables.

**Results:**

The outbreak started from the 4^th^ to the 25^th^ epidemiological week of 2011 with a peak on the 10^th^ week after onset. The attack and case fatality rates were 34/100000 inhabitants and zero respectively. Females and infants aged 9-59 months represented 97(63.4%) and 75(49%) of cases respectively. Bonadiwoto health area alone had 81(52.9%) of cases. Of the 153 cases, only 34(22.2%) had a card-confirmed measles vaccination status. Active community surveillance permitted the identification of 42(27.5%) cases.

**Conclusion:**

Low measles vaccine coverage rate over the past years in the Nylon health district led to the accumulation of susceptible individuals which coupled with poor environmental conditions favoured inter-human spread of measles. Developing novel strategies to vaccinate every child, especially the Hard-to-Reach in the slums of the district will help to prevent future outbreaks.

## Introduction

Measles is an acute highly contagious viral disease with prodromal fever, conjunctivitis, coryza (runny nose), cough and small spots with white or bluish-white centers on an erythematous base on the buccal mucosa (Koplik spots). A rash develops, starting on the face and upper neck and gradually spreading downwards lasting 4 to 7 days and sometimes ends with desquamation [1, 2]. It affects mostly children and the virus is transmitted via droplets from the nose, mouth or throat of infected persons through coughing, sneezing or close personal contact or direct contact with infected nasal or throat secretions. The virus remains active and contagious in the air or on infected surfaces for about two hours. It can be transmitted by an infected person from 4 days prior to the onset of the rash to 4 days after the rash erupts and can be prevented by immunization.

Measles remain a leading vaccine-preventable cause of child mortality worldwide. An estimated 2.1 million people around the world died in 2002 of diseases preventable by widely used vaccines. This toll included 1.4 million children under the age of five. Among these childhood deaths, over 500 000 were caused by measles [3]. Measles is still a public health problem especially in South Asia (highest mortality) and Africa. According to the World Health Organization (WHO), more than 20 million people are affected by measles each year with 95% of measles deaths occurring in low income countries that have weak health infrastructures. In 2008, 164 000 measles deaths were reported worldwide mostly in under 5 children, that is nearly 450 deaths every day or 18 deaths every hour [4].

In order to reduce the morbidity and mortality caused by measles, the Cameroon Ministry of Health organized a mass measles vaccination campaign in 2002 targeting children aged 9 months to 14 years. Before then, measles containing vaccine was administered with routine services in health facilities with a persistently low coverage that varied from 36% in 1990 to 49% in 2000 [5]. There were yearly epidemics in the three northern regions of the country producing over 50% of the measles cases and an epidemic every 2 to 3 years in the other seven regions of the country. With a vaccination coverage rate of 92% during the measles mass campaign, Cameroon was able to reduce the number of measles cases by 98.5% in 2004 [6]. Despite such a significant progress, measles outbreaks of varying intensity have been reported here and there in the country since then [7]. In response to the wave of epidemics in many districts in 2009, a nationwide mass measles vaccination campaign was organized in the same year targeting children aged 9 to 59 months. In 2010, ten measles outbreak were reported in the northern part of the country with over eight hundred cases and a case fatality of 2.2% [8] .

Two health districts in the littoral region (Nylon and Bonassama Health Districts) were involved in the series of measles outbreak in 2009 but these outbreaks were poorly documented or followed up. On the 23rd of February 2011, the regional Delegation of Public Health for the littoral was informed by the Central Technical Group of the Expanded Programme on Immunization (CTG EPI) that, Measles Specific Immunoglobulin M (IgM) antibodies were confirmed at the Centre Pasteur Cameroun reference laboratory in at least three specimens collected and sent to Yaoundé under routine measles surveillance within an interval of four weeks from the Nylon Health District. This is in accordance with the definition for a measles outbreak as stated in the country's Standard Operating Procedures for the Expanded Programme on Immunization (EPI) as well as the WHO guidelines [9, 10]. Nylon Health District was classified as having a measles outbreak. Immediately, the level of alertness within the district as well as the neighbouring districts was raised and strategies set up for proper clinical and data management, reinforcing hospital and community surveillance. We hereby present a description of the measles outbreak in the Nylon Health District in terms of person, time and place.

## Methods

### Setting

Created in 1992, Nylon Health District is one of the six health districts in the city of Douala and the second most populated of the nineteen health districts that constitute the Littoral region. It is an urban slum located in the south eastern part of the city with an estimated population of 444,010 inhabitants in 2011, distributed over 700 hectares of land. It is divided into nine health areas and has 52 health facilities (public, private, confessional), both legal and illegal.

As soon as the regional delegation of public health was informed by the central level on the advent of a measles outbreak in the Nylon Health District in the month of February 2011, they also informed the district. A training session involving health personnel from health facilities within the district was organized by the regional delegation at the district service on how to reinforce surveillance, clinical and data management. During this training, copies of measles line listing forms amongst other documents were handed to the personnel and they were expected to report to the district on daily basis, who in turn reported to the region on Thursdays (or on request). The regional delegation reported to the CTG EPI every Friday of the week.

### Study population and design

It was a retrospective cross sectional study in which all suspected measles cases as per the WHO case definition, registered in the line listing used by the Nylon District Service during the measles outbreak in 2011 were included. The cases were reviewed for information regarding the individuals (age, sex, and vaccination status), place (health area of residence), time (epidemic curve from date of disease onset), clinical factors (signs and symptoms, outcome) and laboratory results.

### Case definition

A suspected measles case was any person living in Nylon Health District who presented to any of the health facilities within the district (or found in the community through active search) from the 27th of January 2011 to the 30th of June 2011 with a Rash and Fever plus at least one of the following symptoms: cough, coryza, conjunctivitis.

### Specimen collection and laboratory analysis

This followed the routine measles surveillance condition whereby 5ml of blood was collected from suspected measles cases received in health facilities within 30 days after the onset of skin rash. Serum was obtained after decanting or centrifuging in a sterile tube labeled with the patient's identification and specimen date collection. The sera were transported to the Centre Pasteur Cameroun Reference laboratory using a vaccine carrier with two ice packs at a temperature of +4 to +8°C alongside the patient's filled measles investigation form. The sera were tested for Measles-specific Immunoglobulin M (IgM) antibody using Enzyme Linked ImmunoSorbent Assay (ELIZA). Only samples negative for measles IgM were tested for Rubella-specific Immunoglobulin M (IgM) antibodies. The results were sent to the CTG EPI which in turn sent feedback to the region. As soon as the epidemic threshold of 3 laboratory confirmed measles IgM positive cases was attained (after collecting 13 specimens) within the Nylon Health District, the district was asked to stop specimen collection as recommended in the EPI Standard Operating Procedures and WHO guidelines and proceeded with putting in place a line listing [9, 10] .

### Data collection

We used the line listing available at the District Service at the end of the outbreak which in itself is a synthesis of the line listing submitted to the district by health facilities during the outbreak. All missing information in the line listing were completed from consultation and admission registers found in the various health facilities where patients were received before the data was analyzed.

### Data analysis

Data was entered and analyzed using Epi Info version 3.5.3 for Windows and Microsoft Office Excel 2010. The line listing was typed in an Excel sheet and imported into Epi info 3.5.3. Frequency tables were ran, missing values and other inconsistencies corrected. Proportions per variable category were estimated and presented in frequency tables. An epidemic curve was drawn using the date of disease onset as declared by the patient or mother (or guardian) in case of infants. The mean age of females and males were compared using the Mann-Whitney test and a P-value < 0.05 was considered significant.

## Results

There were 162 cases in the line listing, 09 of which did not respect the case definition. We therefore present a detailed analysis of 153 suspected measles cases.

### Socio-demographic characteristics of measles cases

Of the 153 cases, 42(26%) were found in the community through active search. The age of cases ranged from 2 months to 31 years with an average of 56.1 ± 63.4 months. The mean age of males (56.1 ± 51.4 months) and females (56.1 ± 69.6 months) were similar (P = 0.283). Infants aged 9 to 59 months 75(49%) were the most affected as opposed to individuals aged 16 years and more 7(4.6%). More than half of the cases live in the Bonadiwoto health area. Females represented 97(63.4%) of the measles cases as shown in Table 1.

**Table 1:** Socio demographic characteristics of suspected measles cases.

Variables	Categories	Frequency	Percentage (%)
Gender	Male	56	36.6
	Female	97	63.4
Age group	<9months	22	14.4
	9-59months	75	49
	5-15yrs	49	32
	>15years	7	4.6
Health Area	Barcelone	14	9.2
	Boko	14	9.2
	Bonadiwoto	81	52.9
	Diboum	7	4.6
	Hors DS	6	3.9
	Ndogpassi Centre	7	4.6
	Ndogpassi ZR	2	1.3
	Ngodi Bakoko	5	3.3
	Oyack	5	3.3
	Soboum	12	7.8
Reporting Unit	Health Facility	111	72.5
	Active Search in the community	42	27.5

ZR : Zone de Recassement.

### Epidemic curve

**Figure 1:**
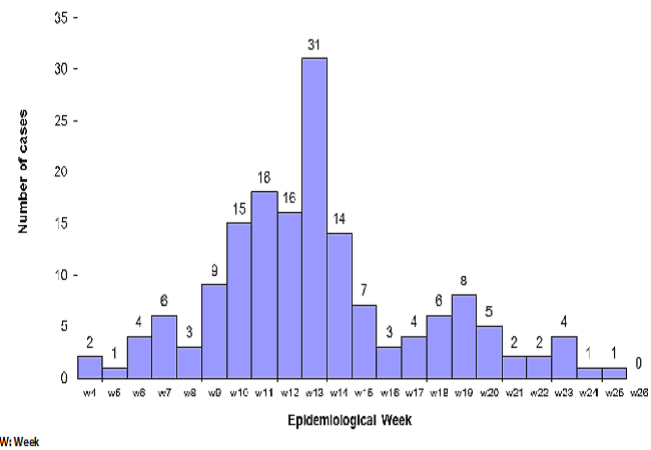
Number of suspected measles cases per week during the outbreak in the Nylon Heath District, Douala Cameroon, from January to June 2011.

Considering the date of disease onset, the outbreak started on the 4^th^epidemiological week with two cases and increased progressively until the 13^th^ week attaining a peak with 30 cases. After the 13^th^ epidemiological week, the number of cases reduced to a trough of two cases on the 16^th^ week. From then onwards, the number of cases started increasing in a monotonic manner to give another smaller peak of 8 cases on the 19^th^ week and ended on the 23^rd^ to 25^th^ week with one case per week as illustrated in Figure 1.

### Clinical and paraclinical characteristics of measles cases

The attack rate was 34/100000 inhabitants. No death was registered. As illustrated in Table 2, all of the 153 measles cases had a notion of fever and skin rash. Cough, conjunctivitis and coryza were present in 136(88.9%), 127(83%), and 121(79.1%) of cases respectively.

**Table 2:** Clinical characteristics of suspected measles cases.

Variables	Categories	Frequency	Percentage (%)
Skin rash	Yes	153	100
	No	0	00
Fever	Yes	153	100
	No	0	00
Cough	Yes	136	88.9
	No	17	11.1
Conjunctivitis	Yes	127	83.0
	No	26	17.0
Coryza	Yes	121	79.1
	No	32	20.9
Skin Rash + Fever + (Cough and/or conjunctivitis and/or Coryza)	Yes	153	100
	No	0	00
Declared Measles vaccination status(All)	Yes (vaccinated)	66	43.2
	No (not vaccinated)	49	32
	Unknown	38	24.8
Objectivated measles vaccination status of the 66 declared vaccinated	Card/booklet	34	51.5
	Said 9 months	14	21.2
	Unknown Date	18	27.3
Declared measles vaccination status of the 75 infants aged 9-59monts	Yes (vaccinated)	38	50.6
	No (not vaccinated)	17	22.7
	Unknown	20	26.7
Objectivated measles vaccination status of the 38 declared vaccinated infants aged 9-59months	Card/booklet	25	65.8
	Said 9 months	4	10.5
	Unknown Date	9	23.7

Of The 153 cases, 66(43.1%) declared to have received at least a dose of measles vaccine. Of the 66 cases, 34(51.5%) could be verified with a vaccination card or hospital booklet. This implies only 34(22.2%) of the 153 cases could be confirmed as haven been vaccinated against measles. Considering infants aged 9 to 59 months, only 25(33.3%) had a verifiable (vaccination card or hospital booklet) measles vaccination status.

Thirteen blood samples were collected from 13 suspected cases during the early phase of the outbreak. Of these, 12 were transmitted to the Centre Pasteur Cameroun reference laboratory at the national level and one was never transmitted. Nine (75%) of the twelve specimens were positive for measles specific Immunoglobulin M (IgM) and three negative. The three negative specimens were also negative for rubella Immunoglobulin M (IgM).

## Discussion

The purpose of this study was to present the epidemiology and clinical picture of the measles outbreak in the Nylon Health District. We found that, Bonadiwoto was the most affected health area. The female gender and infants aged 9 to 59 months represented the bulk of cases. The outbreak which involved mainly unvaccinated individuals lasted 22 weeks with a peak on the 10^th^ week after onset. The case fatality was zero.

Nylon is an urban slum with a very heterogeneous population, spontaneous habitations with a high population density and a high level of promiscuity. It is like the ghetto neighborhood of the economic capital (Douala) of Cameroon. The above conditions are known to favour the transmission of infectious diseases such as measles once the causal germ finds its way in [11, 12]. The district is known to be the entry point of many outbreaks or other health related problems in the cosmopolitan city of Douala just like the concomitant cholera outbreak. The district in general and Bonadiwoto health area in particular harbours most of the neighbourhoods where we find some socio-cultural groups known to be less user friendly with hospital services including childhood vaccination.

The effectiveness of the measles vaccine in reducing the number of measles cases has been established whether in routine situations or in mass campaigns as a response to an outbreak or during supplementary vaccination activities [7, 13]. For this to occur, a high measles vaccine coverage rate needs to be sustained so that susceptible individuals will not go beyond a threshold level for major outbreaks to occur [14]. The Measles Containing Vaccine coverage rate of the Nylon health district was not the best over the last five years. According to unpublished data from the regional delegation of public health for the littoral region, the administrative measles vaccination coverage rate of the district dropped progressively from 80% in 2006 to 62% in 2008 and later increased to 87% and 97% in 2009 and 2010 respectively. This low measles vaccination rate over time must have led to the accumulation of a susceptible population especially amongst the under five children. This surely explains why children aged 9 to 59 months were the most affected. Coupled with the environmental condition described above, once the index case (a child from the northing region of Cameroon where there was an ongoing measles outbreak) introduced the virus into the community, the virus easily found a suitable terrain and other vulnerable persons. This is further illustrated in the epidemic curve, where we see a rapidly spreading infectious disease with a short incubation period and an inter-human transmission pattern.

Due to the quest for a better life, people leaving their villages for the city, have the tendency to settle in Douala, the economic capital of Cameroon. It becomes difficult to master the real population of the Nylon Health District hence the denominator problem as we call it. The present population (just like the previous once), which is an extrapolation from the 2005 general population census still underestimates the population of the Nylon Health District. This is illustrated during mass campaigns (such as poliomyelitis, deworming or vitamin A supplementation) activities where the district usually score an administrative coverage of more than 100% according to unpublished data in the regional EPI unit. Therefore one may be tempted to say that, with the measles vaccination coverage of over 80% in 2009 and 2010, one of the strategies adopted by the World Health Assembly to control measles (attaining at least an 80% measles coverage rate in every health district) has been met in the Nylon health district [15]. Unfortunately, the administrative coverages of 87% and 97% are far below expectations because of the denominator problem hence leaving the population at risk of a measles outbreak.

More so, almost 80% of all the measles cases in the Nylon outbreak (or two thirds of cases aged 9 to 59 months) could be considered as unvaccinated because of unverifiable vaccination status. This brings up many questions that need to be answered in the district such as accessibility to vaccination services, the quality of vaccination and dropouts rates. Similarly, measles outbreaks with similar trends (low vaccination coverage, persons aged less than five years being the most affected) have been reported in other areas such as the Mirriah district in Niger [16] and the Shivpuri district in India [17]. On the other hand, bearing in mind that, a single dose of measles vaccine administered at the age of 9 months as in the EPI programme of Cameroon, is associated with an 85% efficacy [10], some infants with card-confirmed measles vaccination status could still contract the disease. Thus there is a 15% probability that, infants normally vaccinated may not be protected. These infants added to the unvaccinated children increases the number of measles susceptible persons over time.

Almost a quarter of the cases were identified by health workers in the community through active surveillance. These were children treated by their parents using traditional herbs or over the counter drugs. Given the fact that up to a quarter of measles cases were identified in the community, it is likely that some cases must have gone unnoticed by the health system thereby leading to an underestimation of the burden of the outbreak. The parents of cases identified in the community rightly thought of measles but preferred household treatment. The case definition used was that proposed by the WHO and stated in the EPI standard operating procedures [9, 10, 12].

Several control strategies were put in place as soon as the region got information of the outbreak. These were: re-enforcing surveillance and routine vaccination, mass vaccination campaign, information education and communication, proper management of cases and complications. The mass measles vaccination campaign was organized in the high hit health areas on the 14^th^epidemiological week targeting children aged 9 to 59 months. Details of the response are in a follow up paper pending publication.

We reported zero death in our results. Some cases of deaths might have gone unnoticed in the community or unreported by health facilities. Nonetheless, we reviewed hospital registers of all 28 health facilities that reported measles cases for all deaths during the period of the outbreak (4^th^ to the 26^th^ epidemiological week). In all, 42 deaths were registered during the period by four health facilities. The Nylon District Hospital, which is the first line referral unit in the district alone reported 36(85.7%) of the deaths. The most common cause of death was due to HIV related complications 23(54.8%), all of which occurred in the Nylon District Hospital which is also an HIV/AIDS treatment unit. The other causes of death were: still birth 6(14.3%), Severe anaemia 02(4.8%), Malaria 02(4.8%) and Metabolic disorders 02(4.8%). There was a case of death each due to Asthma, Drowning, Drug intoxication and Tuberculosis. None of these patients presented with signs that respected the WHO definition of a suspected case of measles during admission and their medical records were not in favour of measles related complications such as Gastroenteritis, Pneumonia, Sinusitis, Otitis Media, Mouth Ulcers, Upper airway obstruction, Corneal drying, Keratomalacia, Blindness, Malnutrition, Convulsions and Brain damage. Active surveillance helped us to identify 42(27.5%) cases in the community and no community death. This goes to limit misclassification, though the registers of those health facilities which did not report measles cases were not reviewed.

## Conclusion

The suboptimal measles containing vaccine coverage rate in the Nylon Health District led to the accumulation of susceptible individuals which coupled with the poor environmental conditions favoured inter-human spread of measles. Developing strategies to vaccinate each and every child especially the hard to reach in the slums of the Nylon Health District (irrespective of the cause of non vaccination) will help to prevent future outbreaks in the district.
